# The impact of measurement differences on cross-country depression prevalence estimates: A latent transition analysis

**DOI:** 10.1371/journal.pone.0198429

**Published:** 2018-06-07

**Authors:** Pamela Scorza, Katherine Masyn, Joshua A. Salomon, Theresa S. Betancourt

**Affiliations:** 1 Department of Psychiatry, Columbia University Medical Center, New York, New York, United States of America; 2 New York State Psychiatric Institute, New York, NY, United States of America; 3 Department of Epidemiology and Biostatistics, Georgia State University, Atlanta, GA, United States of America; 4 Center for Health Policy, Freeman Spogli Institute for International Studies, Stanford University, Stanford, CA, United States of America; 5 School of Social Work, Boston College, Chestnut Hill, MA, United States of America; Stellenbosch University, SOUTH AFRICA

## Abstract

**Background:**

Depression is currently the second largest contributor to non-fatal disease burden globally. For that reason, economic evaluations are increasingly being conducted using data from depression prevalence estimates to analyze return on investments for services that target mental health. Psychiatric epidemiology studies have reported large cross-national differences in the prevalence of depression. These differences may impact the cost-effectiveness assessments of mental health interventions, thereby affecting decisions regarding government and multi-lateral investment in mental health services. Some portion of the differences in prevalence estimates across countries may be due to true discrepancies in depression prevalence, resulting from differential levels of risk in environmental and demographic factors. However, some portion of those differences may reflect non-invariance in the way standard tools measure depression across countries. This paper attempts to discern the extent to which measurement differences are responsible for reported differences in the prevalence of depression across countries.

**Methods and findings:**

This analysis uses data from the World Mental Health Surveys, a coordinated series of psychiatric epidemiology studies in 27 countries using multistage household probability samples to assess prevalence and correlates of mental disorders. Data in the current study include responses to the depression module of the World Mental Health Composite International Diagnostic Interview (CIDI) in four countries: Two high-income, western countries—the United States (n = 20, 015) and New Zealand (n = 12,992)—an upper-middle income sub-Saharan African country, South Africa (n = 4,351), and a lower-middle income sub-Saharan African country, Nigeria (n = 6,752). Latent class analysis, a type of finite mixture modeling, was used to categorize respondents into underlying categories based on the variation in their responses to questions in each of three sequential parts of the CIDI depression module: 1) The initial screening items, 2) Additional duration and severity exclusion criteria, and 3) The core symptom questions. After each of these parts, exclusion criteria expel respondents from the remainder of the diagnostic interview, rendering a diagnosis of “not depressed”. Latent class models were fit to each of the three parts in each of the four countries, and model fit was assessed using overall chi-square values and Pearson standardized residuals. Latent transition analysis was then applied in order to model participants’ progression through the CIDI depression module. Proportion of individuals falling into each latent class and probabilities of transitioning into subsequent classes were used to estimate the percentage in each country that ultimately fell into the more symptomatic class, i.e. classified as “depressed”. This latent variable design allows for a non-zero probability that individuals were incorrectly excluded from or retained in the diagnostic interview at any of the three exclusion points and therefore incorrectly diagnosed. Prevalence estimates based on the latent transition model reversed the order of depression prevalence across countries. Based on the latent transition model in this analysis, Nigeria has the highest prevalence (21.6%), followed by New Zealand (17.4%), then South Africa (15.0%), and finally the US (12.5%). That is compared to the estimates in the World Mental Health Surveys that do not allow for measurement differences, in which Nigeria had by far the lowest prevalence (3.1%), followed by South Africa (9.8%), then the United States (13.5%) and finally New Zealand (17.8%). Individuals endorsing the screening questions in Nigeria and South Africa were more likely to endorse more severe depression symptomology later in the module (i.e. they had higher transition probabilities), suggesting that individuals in the two Western countries may be more likely to endorse screening questions even when they don’t have as severe symptoms. These differences narrow the range of depression prevalence between countries 14 percentage points in the original estimates to 6 percentage points in the estimate taking account of measurement differences.

**Conclusions:**

These data suggest fewer differences in cross-national prevalence of depression than previous estimates. Given that prevalence data are used to support key decisions regarding resource-allocation for mental health services, more critical attention should be paid to differences in the functioning of measurement across contexts and the impact these differences have on prevalence estimates. Future research should include qualitative methods as well as external measures of disease severity, such as impairment, to assess how the latent classes predict these external variables, to better understand the way that standard tools estimate depression prevalence across contexts. Adjustments could then be made to prevalence estimates used in cost-effectiveness analyses.

## Introduction

Mental and substance use disorders are the leading cause of non-fatal disease burden globally [1,2], and depression is the most prevalent mental disorder [3]. Increasingly, economic evaluations are using data from prevalence estimates like the Global Burden of Disease (GBD) studies to estimate return on investments for services that target mental health [4]. In order to provide the most rigorous assessments, global prevalence estimates need to be as accurate as possible. Cross-national studies have found large differences in estimated levels of depression between countries [5–7]. This could be due in part to environmental factors, including exposure to stressors or traumatic events such as political repression, rapid cultural shifts, socioeconomic deprivation and threat of violence [5]. However, some part of these differences are likely due to variations in the performance of measurement tools, a possibility acknowledged in most cross-national psychiatric epidemiologic studies [5,6,8,9] yet rarely explored. Methodological sources of measurement differences include, but are not limited to, cross-national differences in the expression of standard criteria and differences in the willingness of respondents to admit depressive symptoms in an interview even if they actually do experience them [10].

The initial estimates for the 1993 GBD were strongly critiqued [11,12]—data from a number of unpublished studies were used, with information collected via personal communication, and the WHO African region had extremely low coverage with data on only three out of 46 countries and those data based on small villages or towns with sample sizes less than 1000 [11]. Recent analysis have shown that heterogeneity in the data used to calculate the GBD statistics account for over 50% of the variability in prevalence between studies [13]. In the most recent revisions to the GBD study [14], a modeled estimation included improved data sources [15]. A number of assumptions must be made when including these disparate data in models to arrive at regional prevalence estimates, including how various studies with specific, non-representative populations are weighted; how studies that do not include point-prevalence are converted to be included in the estimates, given that GBD estimates are based on point prevalence; and how estimates from studies using screening tools instead of diagnostic tools are adjusted. Because these assumptions are not explicit in the explication of the model estimates for regional prevalence in the GBD [15], it is difficult to analyze the influence of measurement differences on the actual prevalence estimates used to calculate the global burden of depression.

However, a very well planned and carefully coordinated cross-national study that provides key sources for the GBD estimates—the World Mental Health (WMH) Survey Initiative—provides data that make such an analysis of measurement invariance possible. A surprising finding in the WMH surveys was that Nigeria had an extremely low estimate of prevalence [16], a finding that has caused researchers to question the validity of those data [17]. This paper uses WMH survey data from Nigeria as well as South Africa—the only other sub-Saharan African countries in the WMH surveys—in addition to two high-income western countries—the United States and New Zealand, to examine measurement differences in the WMH Composite International Diagnostic Interview (WMH CIDI) across countries. The analysis uses a latent variable technique to allow for measurement error in the CIDI module, particularly in screening questions and exclusion criteria, and to quantify and compare that measurement error across countries. In doing so we provide an estimate of the impact of these measurement differences on cross-country depression prevalence estimates.

## Methods

### Data/Sample

The WMH CIDI is an adaptation of the World Health Organization (WHO) CIDI for use in WHO’s WMH surveys [18] in 27 countries. Principal investigators from South Africa and Nigeria granted access to the WMH datasets from those countries. World Mental Health Survey data from the United States and New Zealand are publically accessible. The samples from these four countries are described below.

### South Africa

The South African Stress and Health Study (SASHS), which was the South African component of the WMH surveys, used a national probability sample of 4,351 South African adults [19]. Individuals of all race and ethnic backgrounds were included in the study. The sample was selected using a three-stage clustered area probability sample design. The first stage involved the selection of stratified primary sample areas based on the 2001 South African Census Enumeration Areas (EAs). The second stage involved the sampling of housing units within clusters selected within each EA. The third stage involved the random selection of one adult respondent in each sampled housing unit. Further sampling details are described in Williams et al. 2004 [20]. Overall response rate was 85.5%.

### Nigeria

The Nigerian Survey of Mental Health and Well-being used a four-stage area probability sample of adults in households in 21 of Nigeria's 36 states, representing about 57% of the national population. A detailed description of the sampling procedure can be found in Gureje et al. 2006 [21]. In brief, the first stage consisted of sampling 40 local government areas; the second stage selected four census enumeration areas from each primary sampling unit; in the third stage, households were randomly selected from census enumeration areas; and in the fourth stage, a probability procedure was used to select one respondent per household. The surveys were conducted in Yoruba, Igbo, Hausa and Efik languages. Face-to-face interviews were carried out on 6,752 respondents. The overall response rate was 79.3% [16].

### New Zealand

The New Zealand Mental Health Survey, New Zealand’s contribution to the WMH surveys, used a nationally representative sample of 12,992 adults living in permanent private dwellings throughout New Zealand. The response rate was 73.3% [22]. Stratified sampling was used wherein census blocks were selected as primary sampling units from within geographic strata. Households were then systematically selected within census blocks, and one person was selected from each dwelling using a Kish grid. The survey design is described in detail in Wells et al. 2006 [23].

### United States

The US data for the WMH surveys come from the Collaborative Psychiatric Epidemiology Surveys [24], which comprise three separate surveys, the National Comorbidity Survey Replication, the National Survey of American Life, and the National Latino and Asian American Study. The sample for all three surveys consisted of primary sampling units selected with probabilities proportional to size. Response rates for the three surveys were 70.9%, 71.5%, and 75.7% respectively. Interviews were conducted as computer-assisted personal interviews, computer-assisted telephone interviews, and telephone interviews. Data collection for the three surveys was conducted in a total of 252 geographic areas or primary sampling units across the United States. Total sample size was 20,015.

Standardized procedures for interviewer training, translation of study materials and quality control were consistently used in each country of the World Mental Health Surveys [25].

**Fig 1:**
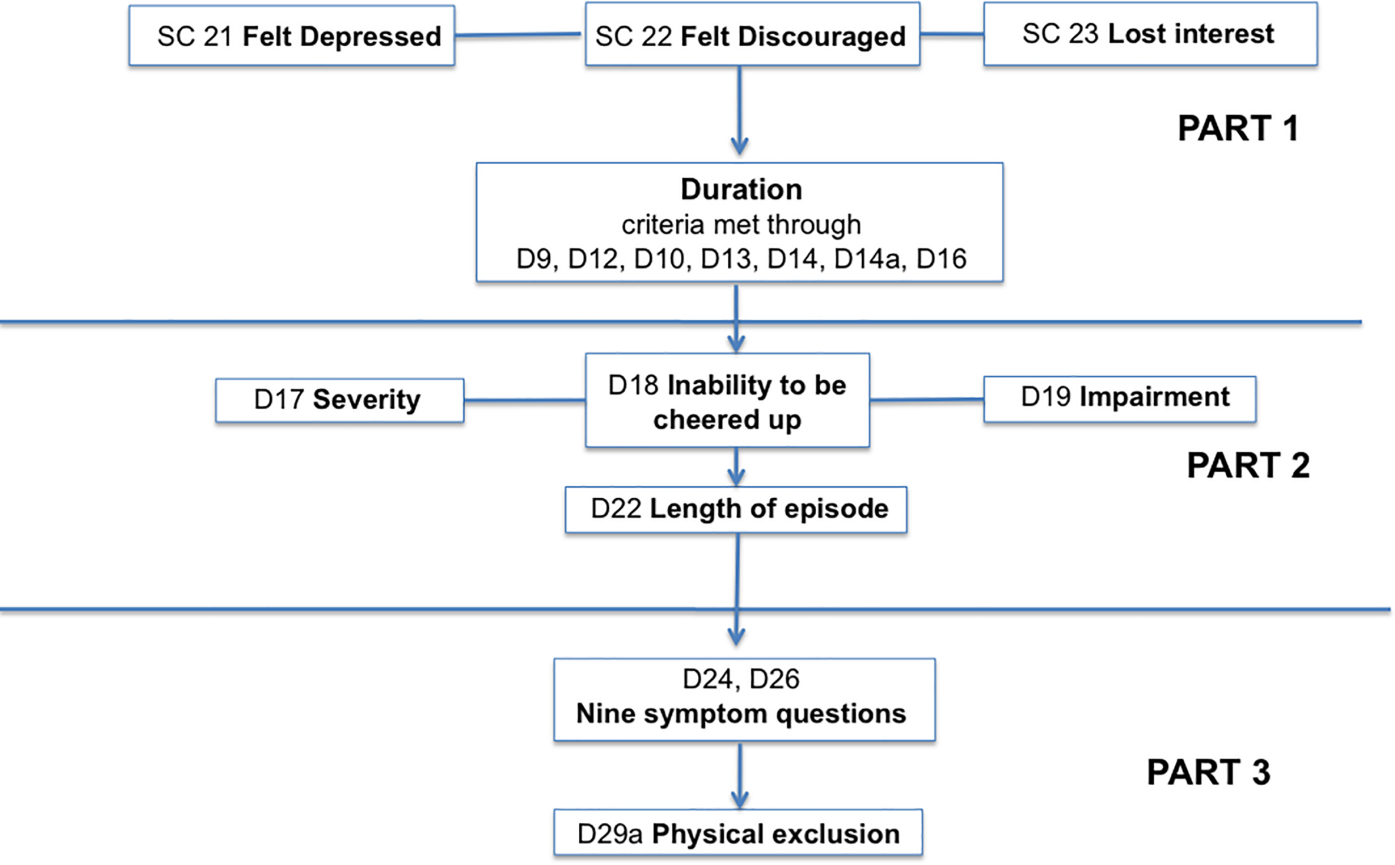
CIDI depression items comprising the three parts of the latent transition model.

Included in this analysis are three dichotomous depression screening items at the beginning of the CIDI that determine whether individuals will be administered the CIDI depression module, as well as all items in the WMH CIDI that are utilized in the DSM-based algorithm for diagnosing depression. Screening questions and CIDI depression module questions were combined into 18 variables, and these variables were divided into three parts based on the points in the module at which large percentages of respondents were excluded from the rest of the module. Fig 1 shows the three parts, and more detailed information is available in S1 File.

## Analysis

Latent class models were fit to each of the three parts of the CIDI depression model in each of the four countries. Latent transition analysis was then applied to model participants’ progression through the CIDI depression model and to estimate the percentage in each country that are classified as “depressed”.

### Latent class models

Latent class analysis (LCA), a type of finite mixture modeling, categorizes respondents into underlying categories (in this case, depressed or not depressed) based on the variation in their responses to questions (in this case the CIDI depression items). A detailed description of applications and recent developments in LCA appear elsewhere [26–30]. LCA was applied to three sequential parts of the CIDI depression module: 1) The initial screening items, 2) Additional duration and severity exclusion criteria, and 3) The core symptom questions. After each of these parts, exclusion criteria expel respondents from the remainder of the diagnostic interview, rendering a diagnosis of “not depressed”. Latent class models were fit to each of the three parts in each of the four countries, and model fit was assessed using overall chi-square values and Pearson standardized residuals. We chose not to rely on the chi-square test for Part 1 because, in general, large sample sizes will cause most chi-square-based statistics to almost always report a statistically significant difference between observed data and model expectations, which suggests model misfit even for a well fitting model. Given the sample size for part one (n~20,000) because no participants have yet been screened out of the module at that point, we rely on residuals to judge model fit rather than chi-square.

Models in which less than 10% of the response pattern residuals were greater than |3| were deemed well-fitting models.

Model selection in Part 1 was limited by the number of parameters. The four dichotomous indicators would only allow for a two class model to be identified [31]. However, because this part of the module (the initial screening questions) excluded a large number of individuals, we wanted to explore additional heterogeneity that may not have been captured in the diagnostic algorithm. We opted, therefore, to use a restricted three-class model in which one class contained those who did not endorse the duration criteria, and the other two classes contained respondents who endorsed one or more screening questions as well as the duration criteria. This restriction created a class that was excluded from the rest of the module in a way that was consistent with the algorithm, i.e. everyone in that class failed to endorse the required duration criteria. For Part 2 (severity and length of episode questions), a similar restriction was placed on a three-class model: one class contained those who did not endorse the episode duration criteria, and the other two classes contained respondents endorsing degrees of severity in addition to the required duration of episodes.

As in part 1, this restriction simultaneously enhanced consistency with the diagnostic algorithm (by creating a class that was excluded by not endorsing the diagnostic criterion of duration) while allowing us to explore additional heterogeneity among those respondents not excluded.

A two-class model was selected for part 3—depressed and not depressed. Because the number of questions allowed for a more exploratory analysis of class structure in Part 3, multiple indices were examined to select a model. The Bayesian Information Criteria (BIC) was used, as well as the log likelihood and Consistent Aikike Information criterion (CAIC) were also examined to assess an optimal number of classes.

Because we had a large enough sample size and because we approached the class enumeration process with some prior latent class model criteria but also wanted to explore additional heterogeneity in the data, we carried out a double split-half validation procedure to assure that the selected models were consistent with the data. Each country’s dataset was first randomly split in half. The class enumeration procedure described above was then conducted on each half of the data separately in each country. The selected latent class model from each half was then fit to the other half of the data, with the threshold parameters and the class-specific item response probability parameters constrained to the values of the parameters in the model from the opposite half. If the model fit of this constrained model was not significantly worse than an unconstrained model for a given half of the data, this indicated that the selected model reflected real heterogeneity in the data and not sample-specific variation, strengthening our confidence in the model. This split half class enumeration and cross-validation procedure was carried out separately with the samples from each of the four countries for each of the three parts of the data.

## Latent transition model

In each of the four countries, a latent transition (LT) model was then specified as described in Scorza et al 2015 [32], with transitions estimated separately between part 1 and part 2 and part 2 and part 3. This was done in each country allowing all parameters to vary between countries.

In order to reliably recover the LCA measurement parameter estimates from the separate LCA measurement models for each part of the module, in the LT model we used random start values for those parameters set equal to the maximum likelihood estimates from the separate part LCAs. Transition restrictions were placed on the LTAs such that individuals who were excluded from the remainder of the module were forced into a “catch” class, so that individuals not contributing information to the module would not influence class structure and transition probabilities. Other transition probabilities were allowed to be freely estimated in each country. Transition probability matrices for parts 1–2 and 2–3 were then multiplied to arrive at transition probabilities from part 1 to part 3 of the module in each country. To estimate depression prevalence in each country, the percentages of individuals in class 3 in part 1 (those who endorsed initial symptom and duration criteria) were multiplied with their conditional probabilities of transitioning to class 2, the “depressed” class, in Part 3. This product was the initial estimate of prevalence. This LT estimate of depression was compared with the estimates based on a straight application of the DSM algorithm.

All models are estimated using the Mplus statistical software package, version 6.0 [33], which allows for the use of random start value perturbations to guard against the algorithm converging to local optima. Complex sampling designs were taken into account- cluster, strata, and individual sampling weights were included in the estimation using the type = complex with estimator = MLR command. This estimation process produces standard errors and chi-square test statistics that are robust to non-normality [34] and standard errors adjusted for non-independence of observations due to clustering, with a post-hoc sandwich estimator. Individual country weights were re-scaled such that their weight in the pooled sample from all four countries was proportional to the relative sample size of the country.

## Results

### Latent class model selection

**Fig 2:**
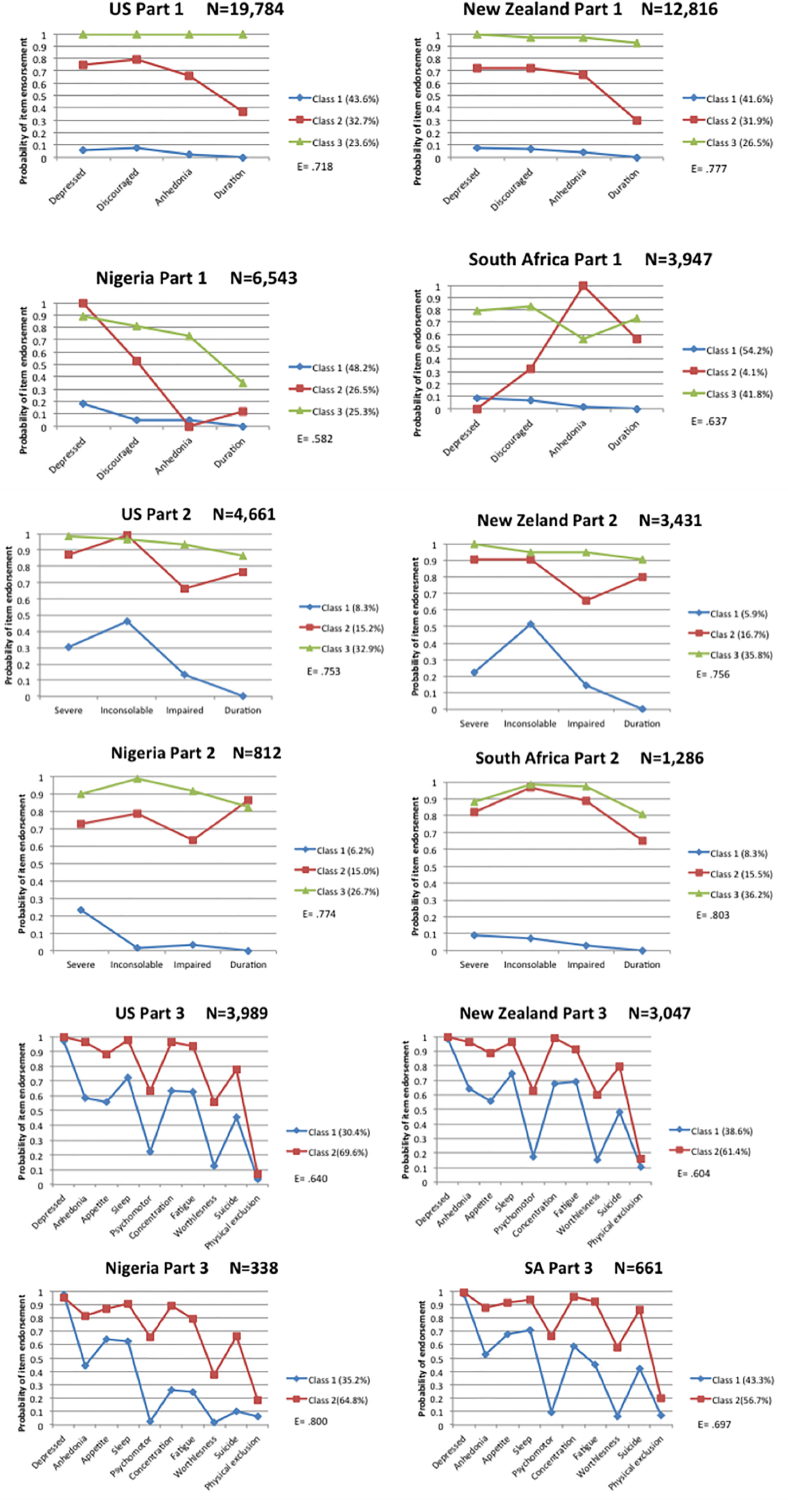
Class-specific item endorsement probabilities for LCA model for each part of the module in four countries. *Note*. Percentages of country samples that are in each class, based on posterior class probabilities, are given in the legends. Entropy (E) is also displayed below the legend.

In each part of the CIDI module, the same latent class model fit the data for each of the four countries. Pearson standardized residuals for observed response patterns indicated good overall fit for the models. All models had less than 10% of the response pattern residuals greater than |3| with the exception of the model for Part 1 in New Zealand, which had 21% of response pattern residuals ≥ |3|, however, no response pattern residuals were >|4|. Chi-square p-values were < .01 for all models in Part 1, which we assume is due to the very large sample sizes in the datasets in this part. Chi-square p-values were 1 for all models in Parts 2 and 3, which have smaller, but still large sample sizes, indicating good fit of the models to the data. The split half validation procedure indicated that the selected model reflected real heterogeneity in the data and not sample-specific variation of this constrained model, given that models selected from one half of the data were not significantly worse fitting than an unconstrained model for the other half of the data, Parts 1–3 in each country. Fit indices for the 2-class model in Part 3 are provided in Table 1. While latent classes need not be ordered, the classes in each country in the three parts do seem to correspond to levels of depression (See Fig 2). Thus while the response probabilities of certain items are different by country, the overall pattern is ordered classes based on likelihood of endorsing depression criteria, which can also be considered depression severity. Relative entropy, a diagnostic index of an LC model’s classification precision, is also shown in Fig 1. Entropy measures posterior classification uncertainty and is bounded between 0 and 1, with 1 being perfect classification precision [35].

**Table 1:** Fit indices comparing models with 2 through 6 classes in part 3 in four countries.

**SOUTH AFRICA**				
n = 309				
# Classes	LL	Chi-2 p-value	BIC	CAIC
**2**	**-1409.401**	**1**	**2939.202167**	**2960.202167**
3	-1394.785	1	2973.036921	3005.036921
4	-1383.883	1	3014.299675	3057.299675
5	-1372.709	1	3055.018429	3109.018429
6	-1362.652	1	3097.971183	3162.971183
**NIGERIA**				
n = 161				
# Classes	LL	Chi-2 p-value	BIC	CAIC
**2**	**-802.731**	**1**	**1712.171492**	**1733.171492**
3	-787.486	1	1737.57694	1769.57694
4	-773.375	1	1765.250388	1808.250388
5	-764.289	1	1802.973836	1856.973836
6	-754.947	1	1840.185284	1905.185284
**USA**				
n = 1898				
# Classes	LL	Chi-2 p-value	BIC	CAIC
**2**	**-7950.452**	**1**	**16059.42368**	**16028.61349**
3	-7880.642	1	16002.83779	15955.88894
4	-7847.658	1	16019.90391	15956.81639
5	-7825.67	1	16058.96202	15979.73584
6	-7805.318	1	16101.29214	16005.92728
**NEW ZEALAND**				
n = 1513				
# Classes	LL	Chi-2 p-value	BIC	CAIC
**2**	**-6657.605**	**1**	**13468.9711**	**17075.16884**
3	-6619.053	1	13472.40519	17092.27919
4	-6600.338	1	13515.51554	17124.11954
5	-6577.317	1	13550.01388	17154.51588
6	-6557.436	1	13590.79223	17191.50423

### Latent transition classification

In Nigeria, prevalence based on the LTA estimate was seven times higher than the estimate based on the DSM algorithm along. Similarly in South Africa, the LTA estimate was one and a half times higher than the estimate based on just the DSM algorithm. On the other hand, in the US and New Zealand the LTA estimates were approximately the same as the DSM algorithm diagnosis. A table of estimates across countries and classification methods is shown in Table 2. Transition probabilities varied between countries, as displayed in Tables 3–5. In South Africa, individuals in class 2 in part 1 were more likely to transition to the more severe class in part 2, as compared with those in the other countries. Individuals in the most severe class in part 1 in Nigeria were much more likely to be in the more severe class in part 2 compared with the other countries. These two differences contribute to the large shifts in prevalence estimates in Nigeria and South Africa from those based on the DSM algorithm to those based on the LTA.

**Table 2:** Depression prevalence estimates across countries by algorithm and latent transition.

	United States	New Zealand	South Africa	Nigeria
DSM Algorithm	13.50%	17.80%	9.80%	3.10%
LTA	12.50%	17.40%	15.00%	21.60%

**Table 3:** Latent transition probabilities across four countries for part 1 to part 2.

**USA**	n = 19734					**NZ**	n = 12816				
		Part 2						Part 2			
		Catch	C1	C2	C3			Catch	C1	C2	C3
Part 1	C1	1	0	0	0	Part 1	C1	1	0	0	0
	C2	0	.113	.814	.073		C2	0	.185	.760	.055
	C3	0	0	.350	.650		C3	0	0	.436	.564
**SA**	N = 3947					**Nigeria**	N = 6543				
		Part 2						Part 2			
		Catch	C1	C2	C3			Catch	C1	C2	C3
Part 1	C1	1	0	0	0	Part 1	C1	1	0	0	0
	C2	0	.205	.669	.126		C2	0	.288	.628	.085
	C3	0	0	.471	.529		C3	0	0	.060	.940

**Table 4:** Latent transition probabilities across four countries for part 2 to part 3.

**USA**	n = 4461				**NZ**	n = 3431			
		Part 3					Part 3		
		Catch	C1	C2			Catch	C1	C2
Part 2	C1	1	0	0	Part 2	C1	1	0	0
	C2	0	.638	.362		C2	0	.682	.318
	C3	0	0	1		C3	0	.081	.919
**SA**	N = 1286				**Nigeria**	N = 812			
		Part 3					Part 3		
		Catch	C1	C2			Catch	C1	C2
Part 2	C1	1	0	0	Part 2	C1	1	0	0
	C2	0	.615	.385		C2	0	.558	.442
	C3	0	.145	.855		C3	0	.084	.916

**Table 5:** Latent transition probabilities across four countries for part 1 to part 3.

**USA**	n = 19734				**NZ**	n = 12816				
		Part 3					Part 3			
		Catch	C1	C2			Catch	C1		C2
Part 1	C1	1	0	0	Part 1	C1	1	0		0
	C2	.113	.519	.368		C2	.185	.532		.292
	C3	0	.223	.778^a^		C3	0	.343		.657^a^
**SA**	n = 3947				**Nigeria**	n = 6543				
		Part 3					Part 3			
		Catch	C1	C2			Catch	C1		C2
Part 1	C1	1	0	0	Part 1	C1	1	0		0
	C2	0	.205	.430		C2	.288	.358		.355
	C3	0	0	.366^a^		C3	0	.112		.888^a^

^a^Shaded cells are those that constitute the latent transition depression diagnosis probabilities.

A result of different transition probabilities across countries, prevalence estimates based on the LT model changes the order of depression prevalence across countries. Based on the DSM algorithm, Nigeria has by far the lowest prevalence (3.1%), followed by South Africa (9.8%), then the United States (13.5%) and finally New Zealand (17.8%). Based on the LT model, Nigeria has the highest prevalence, followed by South Africa, then New Zealand, and finally the US. In addition, the range of depression prevalence between countries is much narrower based on the LT model than it is according to the DSM algorithm. The estimates based on the LT transition span from 15% in South Africa to 21.6% in Nigeria, a range of only 6 percentage points, whereas the range of prevalence estimates based on the DSM algorithm spans from 3.1% in Nigeria to 17.8% in New Zealand, a spread of over 14 percentage points.

## Discussion

This analysis found that depression prevalence estimates across four countries- the United States, New Zealand, South Africa and Nigeria- were markedly different when using a model that allows for the possibility that participants respond differently across countries (the LTA) as compared with one that does not (straight application of the DSM algorithm). Using the LTA, the range of prevalence estimates was narrower across the four countries and the two sub-Saharan African countries had higher prevalence than the United States and New Zealand. Nigeria had the highest prevalence of the four countries when using LTA, while it had the lowest prevalence when using the DSM algorithm alone, and the adjusted difference in prevalence was sevenfold. This finding substantiates skepticism expressed about the accuracy of low levels of depression in Nigeria found in the WMH Surveys (3.1% lifetime prevalence) [17]. Nigeria is one of only two countries represented in the WMH surveys from sub Saharan Africa, so its estimate of depression represents an important contribution to the global epidemiology of depression.

Large differences were found between the four countries in the reporting of screening questions and impairment and duration criteria. Individuals in the two African countries in this study- South Africa and Nigeria- were much less likely to endorse the duration criteria given the same latent class.

Alegria et al. have suggested that screening items might be improperly excluding some individuals from diagnosis in structured diagnostic interviews when used in cross-cultural settings [36]. Yet published validation exercises involving the CIDI have been completed almost entirely in western countries; hence its cross-cultural reliability and validity remain unclear [6,17]. The results show that the impact of these measurement differences on prevalence estimates may be substantial.

Estimates of depression from epidemiologic studies such as the WMH Surveys are particularly important given that these estimates are being used, among other data sources, to estimate depression prevalence and disease burden globally [15]. In addition to using the actual prevalence estimates from CIDI data, structured diagnostic interviews like the CIDI are being used as gold standards on which adjustments to estimates from data using screening tools are based [15]. Information on these adjustments is not transparent in the GBD study, but the results of these analyses suggest that the measurement error, if not taken into account, could affect GBD estimates greatly. GBD estimates, in turn, are being used in models estimating the cost-effectiveness of mental health programs globally [4].

Several limitations should be mentioned. A fundamental concern with the LTA could be that the classes of depression, freely estimated across countries in the LTA, could have different practical and clinical meanings across countries. The meaning of depression categories across cultures is an interesting area that merits further research that blends latent variable measurement invariance analyses with qualitative research to understand local perceptions of the importance of classifications of depression in different contexts. Future analyses using latent classification methods could also use exogenous variables, such as functional impairment, to assess the meaning and comparability of classification across countries. Depending on how they are measured, however, these external variables might also have cross-cultural measurement non-invariance, making their use not unproblematic.

Finally, it should be reiterated that not all differences in prevalence between countries are likely due to measurement differences. As noted by Gureje et al., the age distribution of respondents in different surveys may be a factor. In Nigeria, for example, the majority of the WMH Survey respondents in the country’s survey were younger than the median age of onset of the depression. [37] Indeed, another study in Nigeria found high rates of both prevalence and incidence were reported among elderly persons, aged 65 years and over, in the community. [38] The results of this current study are not meant to suggest that a uniformly similar prevalence would indicate validity of surveys for mental disorders. The results do suggest, however, that a non-insignificant portion of the differences are likely due to measurement non-invariance across countries.

Above all, the analysis in this paper suggests that prevalence estimates from structured diagnostic interviews in radically different settings where measurement properties of tools have not been thoroughly assessed, should not be used uncritically. The data available in the WMH survey provide rich opportunities for understanding cross-cultural measurement of mental disorders more comprehensively. The findings in this paper aim to stimulate more in-depth research on measurement differences. When such a body of research is developed, the field of global mental health will be better positioned to both modify tools for future studies and to use adjusted prevalence estimates from current data sources in cost-effectiveness analyses and resource allocation decisions.

## Supporting information

S1 File Detailed description of items and coding for three parts of the latent transition model. (PDF)
