# Supplementary material for: The impact of measurement differences on cross-country depression prevalence estimates: A latent transition analysis
Source: PLoS One. 2018 Jun 7;13(6):e0198429. doi: 10.1371/journal.pone.0198429 (PMC5991686; doi:10.1371/journal.pone.0198429)
Supplement: S1 File — (PDF) [file pone.0198429.s001.pdf]

SUPPLEMENTARY MATERIAL- DETAILED DESCRIPTION OF ITEMS AND CODING FOR THREE PARTS OF THE LATENT TRANSITION MODEL

<u>Part 1:</u>

The first major cluster of these exclusionary items consisted of the three screening symptoms and the question asking about the duration criterion for experiencing these screening symptoms. In the screening portion of the CIDI, three questions about depression were asked. Endorsing none of the three screening questions ruled an individual out from receiving the depression module in the CIDI. The screening questions asked *"Have you ever in your life had a period lasting several days or longer when most of the day you […]"* the three symptoms being:

1- *Felt sad, empty or depressed* (SC21)

2- Were very discouraged about how things were going in your life (SC22)

3- Lost interest in most things you usually enjoy like work, hobbies, and personal relationships (SC23)

In Nigeria and South Africa, if an individual endorsed the first screening item, the second and third screening items were not asked- the individual was taken directly to the module. Likewise, if the individual endorsed the second screening item, the third was not asked. However, when an individual screened into the CIDI module, the screening symptoms that were not asked if the individual endorsed the first or second screening item were then asked in the module (D1 and D2). Thus, in the creation of the variables for part 1 of the latent transition module of the CIDI, the screening items were coded as positive if the individual endorsed either the question in the screening section or the corresponding screening symptom in the module. This allowed for endorsement of the screening items to be captured in all four countries.

The next possible point of exclusion in the CIDI module was the duration of having experienced the screening symptom(s). The initial duration question asks whether the endorsed screening symptom(s) lasted most of the day nearly every day for two weeks or longer (D9 or D12, depending on which screening items were endorsed). However, if this duration was not endorsed, the individual could still be allowed to proceed through the module if they had a year or more when just about every month they had an episode of experiencing the endorsed symptom(s), each of which lasted several days or longer (D10 or D13 and D14 and D14a). Regardless of whether the individual endorsed the duration of two weeks or the frequent episodes of three days or more, they were then asked about the duration, in hours, of these episodes (D16). If the episodes lasted less than an hour, the individual was excluded from the rest of the module. A variable was created for the screening symptom duration criteria, WEEKS2, and if the individual met these initial conditions, either the two weeks or the year of monthly episodes of at least three days, and at least an hour per episode, this variable was coded one. The three screening questions and this duration criteria formed the indicators for a latent class model labeled “part 1”.

<u>Part 2:</u>

Proceeding through the module, three questions are then asked about the episodes-

1- How severe the emotional distress was (D17)

2- How often the emotional distress was so severe that nothing could cheer up the individual (D18)

3- How often the emotional distress was so severe that the individual could not carry out their daily activities (D19)

These three items have likert scale response options- *mild, moderate, severe,* and *very severe* for the first and *often, sometimes, rarely* and *never* for the second and third. If the individual answers *mild* for the first and *never* for the second and third, they are excluded from the module. Individuals who answer in any other combination are then asked the following question:

"People with episodes of being (sad/or/discouraged/or/uninterested) often have other problems at the same time. These include things like changes in sleep, appetite, energy, the ability to concentrate and remember, feelings of low self-worth, and other problems. Did you ever have any of these problems during one of your episodes of being (sad/or/discouraged/or/uninterested)?
(D21)

Individuals who answer *no* to this question, are excluded from the rest of the module. The remainder were then asked how long the worst episode lasted (D22b). If the worst episode can't be recalled, the module asks the duration of the last bad episode (D22d). The duration of this episode was two weeks or greater, it met the duration criterion for major depressive episode, LEPISODE. The three items about severity of the episodes and the one about the duration of the worst/last bad episode, which is only asked if the presence of other problems is endorsed, formed the indicators for a latent class model labeled "part 2".

<u>Part 3:</u>

Individuals who make it through the questions that comprise parts 1 and 2 in our model proceed through the remainder of the CIDI depression module. To create the latent class indicators for part 3 we consulted the algorithm used to diagnose depression in the Collaborative Psychiatric Epidemiologic Survey (CPES) in the United States in 2001-2003

to ascertain the criteria necessary for diagnosis of major depressive episode. There are five main criteria, labeled A-E in the algorithm. These criteria were operationalized in the formation of latent class indicators for "part 3" of the latent transition model in the following way:

Criterion A:

Criterion A consists of two parts, both of which must be met in order to be diagnosed positively with major depressive episode. Part 1 requires the symptoms to have been present during the same two week period, at least one of the symptoms being either depressed mood or loss of interest or pleasure. The two week criterion is already met by anyone who has reached part 3 because of an answer of at least two weeks D22b or D22d in part 2. Presence of one of the two core symptoms is met by endorsement of any of D24a-D24e. These are included in the part 3 indicators DEPMOOD and LOSSINT. Part 2 of criterion A requires that at least 5 of nine symptoms be present. These nine symptoms comprise nine indicators in the "Part 3" latent class model, coded dichotomously as 1 if any of the included items are endorsed and 0 otherwise. They are:

1- Depressed mood- D24a or D24b or D24c or D24d- DEPMOOD

2- Markedly diminished interest or pleasure- D24e or D24f- LOSSINT

3- Significant weight change or change in appetite- D26a or D26b- APPETITE

4- Insomnia or hypersomnia- D26g or D26h- SLEEP

5- Psychomotor agitation or retardation as noticed by others- D26m or D26o PSYCHOMO

6- Fatigue or loss of energy- D26j - FATIGUE

7- Feelings of worthlessness or excessive or inappropriate guilt- D26v- WORTHLES

8- Diminished ability to think or concentrate, or indecisiveness- D26p or D26r or D26s - CONCENT

9- Recurrent thoughts of death, recurrent suicidal ideation without a specific plan, or a suicide attempt or a specific plan for committing suicide- any of D26aa-D26ee - SUICIDE

Criterion B:

The symptoms do not meet criteria for a Mixed Episode. Because this criterion was not operationalized in the CIDI depression diagnosis, it was not included in this latent class model.

Criterion C:

The symptoms cause clinically significant distress or impairment in functioning. Given that all individuals who made it to Part 3 in the latent transition model already endorsed at least moderate severity or impairment (D17-D19), this criterion is already included in the indicators in part 2.

Criterion D:

The symptoms are not due to the direct physiological effects of a substance, or are not due to a general medical condition- D29a - PHYSEXC

Criterion E:

Bereavement exclusion criterion- 23a ≠ 3. In the CPES, this indicator was not included in the questionnaire, so it is not operationalized in the LC models.

The criteria discussed above which led to the formation of indicators for three parts of a latent transition model cover the criteria for major depressive episodes as operationalized by the scoring algorithm in the CPES. Major depressive disorder with hierarchy requires several other conditions, most of which are not operationalized in the CPES. However, the condition of not having a manic or hypomanic episode was

operationalized. Individuals with manic episodes would be excluded from major depressive disorder with hierarchy as long as the manic episode(s) were not substance or treatment induced or due to the direct physiological effects of a general medical condition. However, given that prevalence of manic episodes in the 12-months prior to the survey in the US was found to be only 1.3% [11], diagnosis of major depressive episode is a proxy for diagnosis of lifetime major depressive disorder categorization as operationalized in the US CPES using the WMH CIDI.
